# Impact of corticosteroid therapy on the outcomes of hepatocellular carcinoma treated with immune checkpoint inhibitor therapy

**DOI:** 10.1136/jitc-2020-000726

**Published:** 2020-10-07

**Authors:** David J Pinato, Ahmed Kaseb, Yinghong Wang, Anwaar Saeed, David Szafron, Tomi Jun, Sirish Dharmapuri, Abdul Rafeh Naqash, Mahvish Muzaffar, Musharraf Navaid, Uqba Khan, ChiehJu Lee, Anushi Bulumulle, Bo Yu, Sonal Paul, Petros Fessas, Neil Nimkar, Dominik Bettinger, Hannah Hildebrand, Tiziana Pressiani, Yehia I Abugabal, Nicola Personeni, Yi-Hsiang Huang, Jingky Lozano-Kuehne, Lorenza Rimassa, Celina Ang, Thomas U Marron

**Affiliations:** 1 Department of Surgery and Cancer, Imperial College London, London, UK; 2 Department of Gastrointestinal Medical Oncology, The University of Texas MD Anderson Cancer Center, Houston, Texas, USA; 3 Department of Gastroenterology, Hepatology & Nutrition, University of Texas MD Anderson Cancer Center, Houston, Texas, United States; 4 Division of Medical Oncology, Kansas University Cancer Center, Kansas City, Kansas, USA; 5 Department of Medicine, Division of Hematology/Oncology, Tisch Cancer Institute, Mount Sinai Hospital School of Medicine, New York, New York, USA; 6 National Cancer Institute, Bethesda, Maryland, USA; 7 Division of Hematology/Oncology, East Carolina University, Greenville, North Carolina, USA; 8 Division of Hematology and Oncology, Weill Cornell Medicine/New York Presbyterian Hospital, Ithaca, New York, USA; 9 Division of Gastroenterology and Hepatology, Department of Medicine at Taipei Veterans General Hospital and Institute of Clinical Medicine, National Yang-Ming University, Taipei, Taiwan; 10 Brody School of Medicine at East Carolina University, Greenville, North Carolina, USA; 11 Surgery and Cancer, Imperial College London, London, UK; 12 Department of Medicine II, Faculty of Medicine, Medical Center University of Freiburg, Freiburg, Baden-Württemberg, Germany; 13 Medical Oncology and Hematology Unit, Humanitas Cancer Center, Humanitas Clinical and Research Center, IRCCS, Via Manzoni 56, Rozzano, Milan, Italy; 14 Department of Biomedical Sciences, Humanitas University, Via Rita Levi Montalcini 4, 20090 Pieve Emanuele, Milan, Italy, Rozzano, Lombardia, Italy

**Keywords:** liver neoplasms, immunomodulation

## Abstract

The impact of corticosteroid therapy (CT) on efficacy of immune checkpoint inhibitors (ICI) is undefined in hepatocellular carcinoma (HCC). We evaluated whether CT administered at baseline (bCT) or concurrently with ICI (cCT) influences overall (OS), progression-free survival (PFS) and overall response rates (ORR) in 341 patients collected across 3 continents. Of 304 eligible patients, 78 (26%) received >10 mg prednisone equivalent daily either as bCT (n=14, 5%) or cCT (n=64, 21%). Indications for CT included procedure/prophylaxis (n=37, 47%), management of immune-related adverse event (n=27, 35%), cancer-related symptoms (n=8, 10%) or comorbidities (n=6, 8%). Neither overall CT, bCT nor cCT predicted for worse OS, PFS nor ORR in univariable and multivariable analyses (p>0.05). CT for cancer-related indications predicted for shorter PFS (p<0.001) and was associated with refractoriness to ICI (75% vs 33%, p=0.05) compared with cancer-unrelated indications. This is the first study to demonstrate that neither bCT nor cCT influence response and OS following ICI in HCC. Worse outcomes in CT recipients for cancer-related indications appear driven by the poor prognosis associated with symptomatic HCC.

## Introduction

Hepatocellular carcinoma (HCC) is at the focus of intense research efforts for the development of immune checkpoint inhibitors (ICI).^1^ Targeted inhibition of programmed cell death (PD-1) receptor/ligand (PD-1/PD-L1) interaction results in measurable anti-tumor responses in a fraction of patients with advanced HCC, a finding that led to the breakthrough approval of nivolumab^2^ and pembrolizumab^3^ by the Food and Drug Administration in light of the results of small, single-arm open-label studies. However, evidence of initial activity has not translated into statistically significant survival benefit in randomized controlled studies of anti-PD-1 monotherapy, a finding that has instigated the clinical development of immunotherapy combinations with anti-cytotoxic T-lymphocyte antigen 4 (CTLA-4) inhibitors^4^ and anti-angiogenics to further enhance the anti-tumor immunity.^5^

Evolving experience in the use of ICI suggests iatrogenic factors as important contributors in shaping clinical responses to immunotherapy.^6 7^ Corticosteroid therapy (CT) is often indicated in the management of cancer-related symptoms such as cachexia, anorexia, central nervous system edema or pain^8^ and is recommended by guidelines as first-line therapy for most immune-related adverse events (irAEs).^9^ CT exerts T-cell suppressive properties by reducing the proliferative potential of naïve T cells^10^ and stimulate regulatory T-cell development.^11^

Immunosuppressive CT may therefore adversely influence outcome in patients receiving ICI^12^ and in fact patients receiving chronic steroid therapy have been for this reason excluded from clinical trials of ICI. The effect of CT either at the moment of ICI commencement or during the course of ICI treatment remains undefined in HCC,^13^ a point of greater consequence in a disease where ICI monotherapy has struggled to demonstrate evidence of sustained clinical benefit. In this global observational study, we sought to document prevalence of and indications for CT use in patients with HCC being treated with ICI, and to examine the relationship between CT exposure and outcome.

## Patients and methods

**Figure 1:**
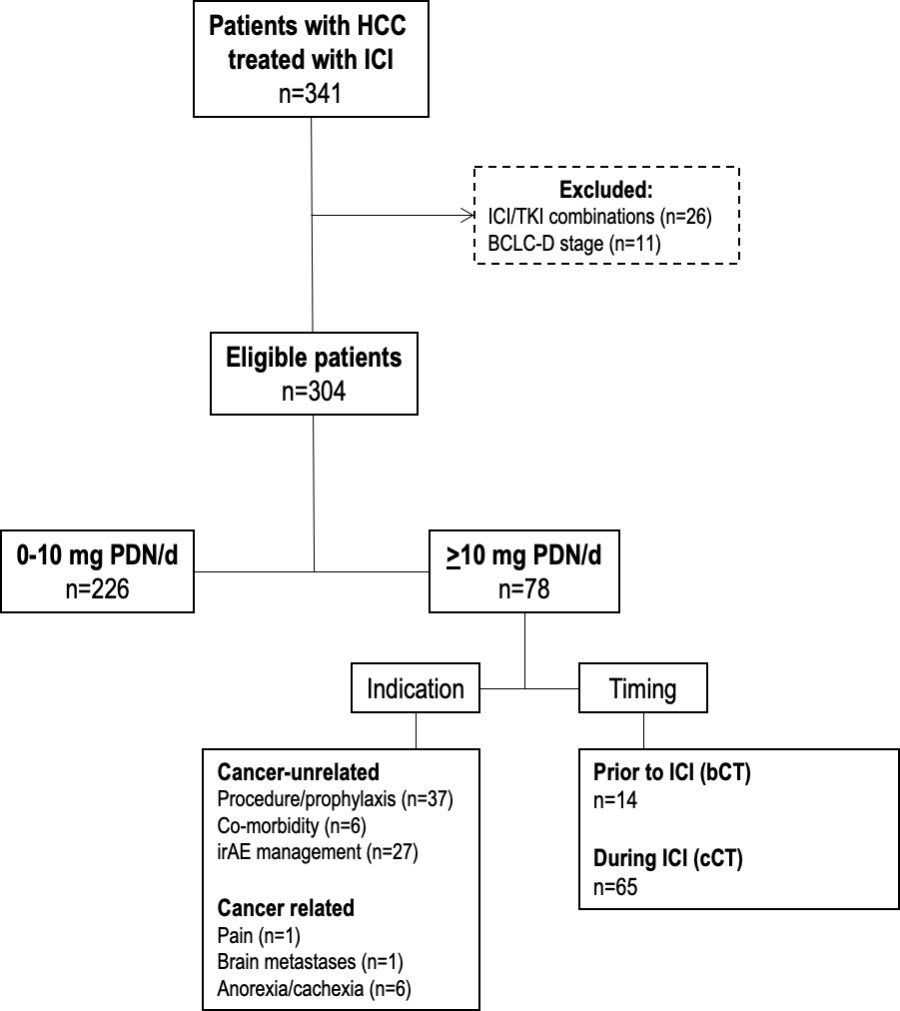
Study diagram of 341 patients with hepatocellular carcinoma (HCC) treated with immune checkpoint inhibitors (ICI). BCLC-D, Barcelona Clinic Liver Cancer stage D; bCT, baseline corticosteroid therapy; cCT, concomitant corticosteroid therapy; irAE, immune-related adverse event; PDN, Prednisolone.; TKI, Tyrosine Kinase Inhibitors.

We established a global dataset of 341 patients with HCC treated with ICI between 2016 and 2019 in nine tertiary referral centers in the USA (n=226), Europe (n=68) and Taiwan (n=47) (online supplemental table S1). Patients with a histological or radiologic diagnosis of HCC based on European Society for the Study of the Liver criteria^14^ and undergoing treatment with checkpoint inhibitors were identified from Oncology Pharmacy electronic records and entered into a prospectively maintained database. In total, we excluded 26 patients who were treated with PD-1/PD-L1 inhibitors in combination with kinase inhibitors and a further 11 patients that classified within Barcelona Clinic Liver Cancer (BCLC) stage D due to performance status 3 and/or Child-Pugh C cirrhosis. This resulted in 304 patients eligible for primary analyses (figure 1).

10.1136/jitc-2020-000726.supp1 Supplementary data

Evaluation of corticosteroid exposure was defined based on timing of administration in line with published literature on the topic.^12^ Patients were defined as receiving CT at baseline (bCT) if administered >10 mg of prednisone (or equivalent) for >24 hours within 30 days prior to ICI. Concomitant corticosteroid therapy (cCT) was defined as stated previously from the day of commencement of ICI until permanent cessation of immunotherapy.

Clinicopathologic variables including overall survival (OS) and progression-free survival (PFS) were derived from electronic medical records. OS was calculated from the date of ICI commencement until last follow-up or patients’ death. Response to ICI was evaluated according to RECIST criteria (version 1.1) and best responses to ICI were recorded for each evaluable patient. Electronic medical records were reviewed to identify prescription of oral or intravenous corticosteroid therapy (CT).

## Statistical analysis

Patient characteristics are summarized as means or medians as appropriate. We conducted analysis of proportions across groups using Pearson χ^2^ or Fisher’s exact tests. We represented group-specific OS and PFS using the Kaplan-Meier curve method and formally evaluated the difference in median survival times between pre-specified groups using the log-rank test. We performed univariable and multivariable analyses of survival using Cox regression models to evaluate the impact of CT on patients’ OS and PFS. To avoid collinearity bias, we evaluated features relating to corticosteroid therapy including overall CT exposure, timing and indication for CT therapy in separate multivariable models as outlined before.^15^ Post-landmark analysis was done in the subset of patients who received corticosteroid (prednisone >10 mg) during ICI treatment. This analysis compared the PFS and OS in patients with/without response with the ICI treatment with cortiscosteroid (CT). Kaplan-Meier analysis was used to estimate the PFS and OS from the time of ICI initiation in patients with (CR+PR) and without (SD+PD) response at 3, 6, 9 and 12 months. Patients who were not evaluable for response at a timepoint were excluded.

All statistical analyses were performed using SPSS V.25.0 and Stata V.16.1 (StataCorp LLC, College Station, TX, USA), and GraphPad Prism (GraphPad Software, La Jolla, CA, USA) with all estimates being reported with corresponding 95% CIs and a two-tailed level of significance of p<0.05.

## Results

### Patients’ demographics and treatment characteristics

Out of 304 eligible patients in the study, the majority were cirrhotic (n=217, 71%) due to hepatitis C infection (n=119, 39%). All patients had measurable disease based on RECIST 1.1 criteria at ICI commencement. Baseline tumor staging according to Barcelona Clinic Liver Cancer (BCLC) algorithm showed most patients to qualify criteria for stage C disease (n=230, 76%).

In total, 273 patients (90%) had received prior therapy for HCC and 179 (59%) were sorafenib experienced. During the observation period, the vast majority of patients (n=279, 92%) received single-agent anti-PD(L)−1 ICI, whereas 25 received combined PD-1/CTLA-4 ICI (8%).

Evaluation of radiologic response to treatment based on RECIST 1.1 criteria (investigator assessed) demonstrated an ORR of 20%, with 23 complete (7.5%) and 38 partial responses (12.5%). Ninety-nine patients (33%) achieved disease stabilization, whereas 127 (42%) were ICI refractory, defined as progressive disease at first imaging reassessment. After a median follow-up of 10.3 months (95% CI 10.6 to 13 months), 153 patients had died (51%) and all but 72 (24%) had discontinued ICI therapy, mostly due to disease progression (n=144, 48%). In total, 113 patients (37%) experienced at least one treatment-related adverse event (AE), 57 of which (18%) were of grade >2 according to Common Terminology Criteria for Adverse Events (version 5.0). Permanent discontinuations due to unacceptable toxicity occurred in 11 patients (4%). Median OS from ICI commencement was 12.3 months (95% CI 9.8 to 15.4 months), median ICI duration was 3.7 months (IQR 6) and median PFS was 6 months (95% CI 4.6 to 8.2 months).

### Corticosteroid therapy

CT was prescribed to 78 patients (26%), defined as bCT in 14 (5%) and cCT in 64 (20%). Two patients (1%) received CT prior to and during ICI: one patient who received CT for palliation of cancer-related symptoms was ascribed to the bCT group. A second patient who received CT as a single course 7 days prior to ICI for intravenous iron infusion and three subsequent courses during ICI was ascribed to the cCT group. Timing of corticosteroid exposure was not associated with HCC stage, liver function and performance status (table 1). Neither bCT (p=1.0) nor cCT (p=0.62) were associated with line of therapy. The most frequent indication for bCT was peri-procedural/prophylactic therapy (n=12, 73%) in patients who received loco-regional therapy within 30 days from ICI initiation (n=9) or who required CT cover for contrast allergy during imaging studies (n=3). Conversely, irAE management was the most frequent indication for cCT (n=27, 44%, p=0.003; online supplemental table S2). Median duration of CT was 4 days (IQR 13) and median daily dose of prednisone equivalent was 50 mg (IQR 30). CT duration was longer in the cCT compared with bCT group (median 6 (IQR 13) vs 2 (IQR 2) days, p=0.006), whereas daily prednisone dose was comparable (median 50 (IQR 40) vs 50 (IQR 22.5) mg/day, p=0.09; online supplemental tables S3–4).

**Table 1:** Clinicopathologic features of the patient population

Characteristic	All patients n=304 (%)	Corticosteroid exposure			P value*
		Prednisone 0 to <10 mg n=226 (%)	Prednisone >10 mg prior to ICI n=14 (%)	Prednisone >10 mg during ICI n=64 (%)	
Gender					0.68
Male	235 (77)	172 (76)	11 (79)	52 (82)	
Female	69 (23)	54 (24)	3 (21)	12 (18)	
Cirrhosis					0.23
Present	216 (71)	156 (69)	9 (64)	51 (80)	
Absent	87 (29)	69 (31)	5 (36)	13 (20)	
Unknown	1 (0.3)	1 (0.4)	0 (0)	0 (0)	
Etiology of chronic liver disease					0.85
Viral	196 (65)	144 (64)	10 (71)	42 (66)	
Non-viral	106 (34)	80 (35)	4 (29)	22 (34)	
Unknown	2 (0.7)	2 (0.9)	0 (0)	0 (0)	
Child-Turcotte-Pugh Class					0.6
A	225 (74)	170 (75)	9 (64)	46 (72)	
B	79 (26)	56 (25)	5 (36)	18 (28)	
Barcelona Clinic Liver Cancer stage					0.35
A–B	74 (24)	51 (23)	3 (21)	20 (31)	
C	230 (76)	175 (77)	11 (79)	44 (69)	
Maximum diameter of largest lesion (cm)					0.96
Median (IQR)	5.5 (5.5)	5.5 (6)	5 (-)	5 (4.5)	
n	202	166	1	35	
Metastatic sites					0.81
0–1	166 (55)	136 (60)	1 (7)	29 (76)	
>2	45 (15)	36 (16)	0 (0)	9 (9)	
Unknown	93 (31)	54 (24)	13 (93)	26 (41)	
Prior systemic therapy for HCC					0.66
0–1	277 (81)	204 (90)	13 (93)	60 (94)	
>2	27 (9)	21 (10)	1 (7)	4 (6)	
Immunotherapy treatment					0.1
Monotherapy	279 (92)	204 (82)	14 (100)	61 (91)	
Combination	25 (8)	22 (18)	0 (0)	3 (9)	
ECOG Performance Status					0.46
0–1	197 (65)	162 (72)	1 (7)	34 (53)	
≥2	13 (4)	9 (4)	0 (0)	4 (6)	
Unknown	94 (31)	55 (24)	13 (93)	26 (41)	
Alfa-fetoprotein					0.42
<400 ng/mL	178 (59)	129 (57)	10 (71)	39 (61)	
>400 ng/mL	118 (39)	93 (41)	4 (29)	21 (33)	
Unknown	8 (3)	4 (2)	0 (0)	4 (6)	
Albumin					0.25
Median (IQR)	36 (9)	36 (8)	33 (10)	36 (10)	
n	298	220	14	64	
Bilirubin					0.64
Median (IQR)	14 (12)	14 (12)	14.5 (12)	15 (10)	
n	302	224	14	64	
Platelet count					0.58
Median (IQR)	160 (119)	160 (120)	100 (–)	162 (92)	
n	210	171	1	38	

*Excludes the “Unknown” category in the statistical test.

ECOG, Eastern Cooperative Oncology Group; HCC, hepatocellular carcinoma; ICI, immune checkpoint inhibitors.

### Impact of CT on clinical outcomes from ICI

**Figure 2:**
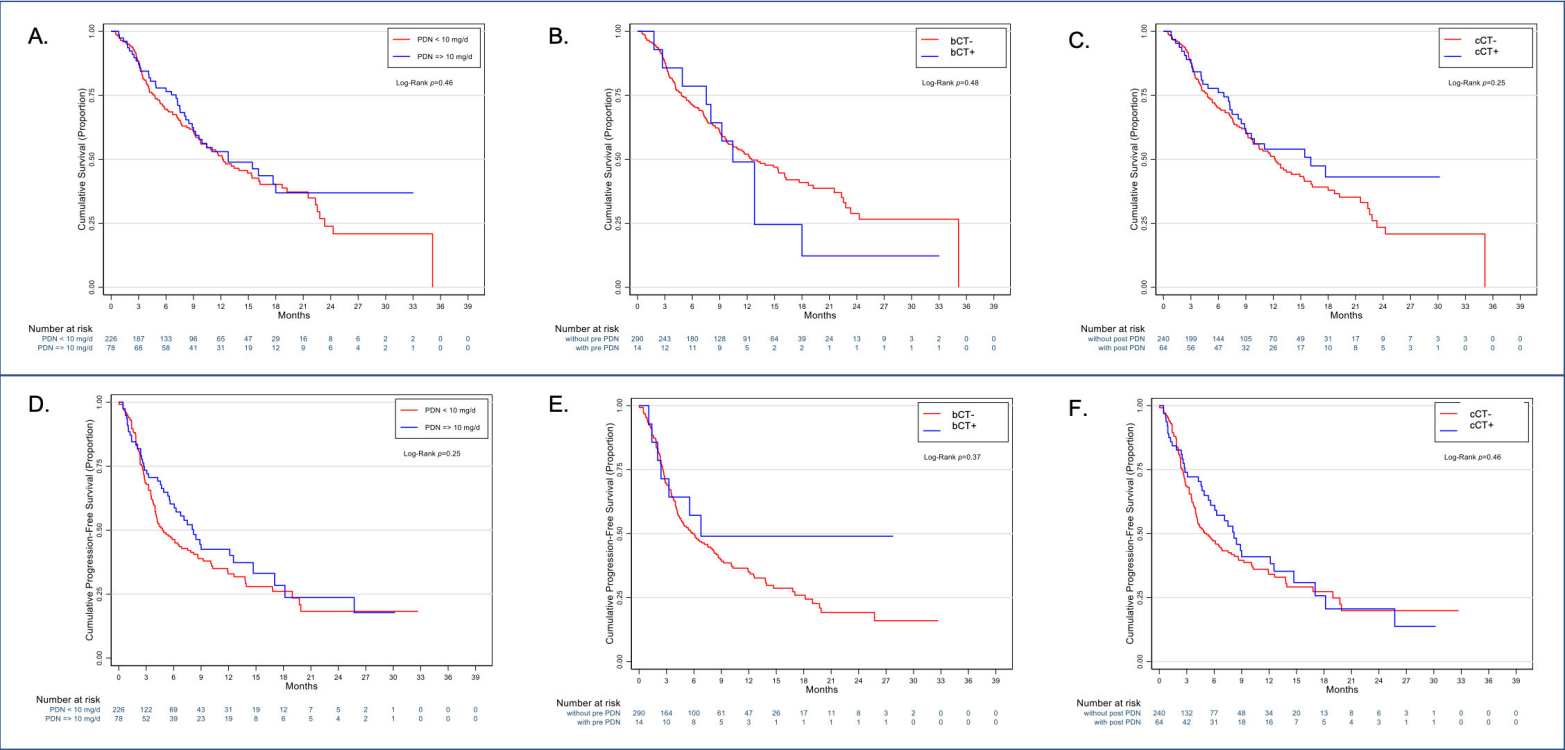
Exposure to corticosteroid therapy (CT, panel A) and timing of CT (panels B, C) do not influence the overall survival and progression-free survival (panels D, E, F) of patients with hepatocellular carcinoma receiving immune checkpoint inhibitors.

The median OS of patients exposed to >10 mg of prednisone at any point throughout the study was 12.8 months (95% CI 9 to NR) and not different from the 12.2 months (95% CI 9.7 to 16.1, p=0.50) observed for patients who received 0 to <10 mg. Median PFS (8.2 months, 95% CI 5.5 to 12.5 vs 4.8 months, 95% CI 4.0 to 7.5 months, p=0.25) and ORR (21.3% vs 21.1%, p=0.96) were also similar between groups. When compared with bCT-unexposed (median OS 12.2, 95% CI 8.7 to 15.6) or cCT-unexposed patients (median OS 11.7, 95% CI 9.4 to 13.9), neither bCT (median OS 10.4, 95% CI 7.5 to 13.3, p=0.53) nor cCT exposure (median OS 16.1, 95% CI 8.8 to 22.5) was indicative of worse OS (figure 2A–C). No difference in median PFS was observed across bCT (6.7 months, 95% CI 1.0 to 13.0 months vs 5.8 months, 95% CI 4.0 to 7.6, p=0.36), cCT (8.1 months, 95% CI 5.5 to 10.7 months vs 4.9 months, 95% CI 3.3 to 6.5, p=0.42; figure 2E, F).

**Figure 3:**
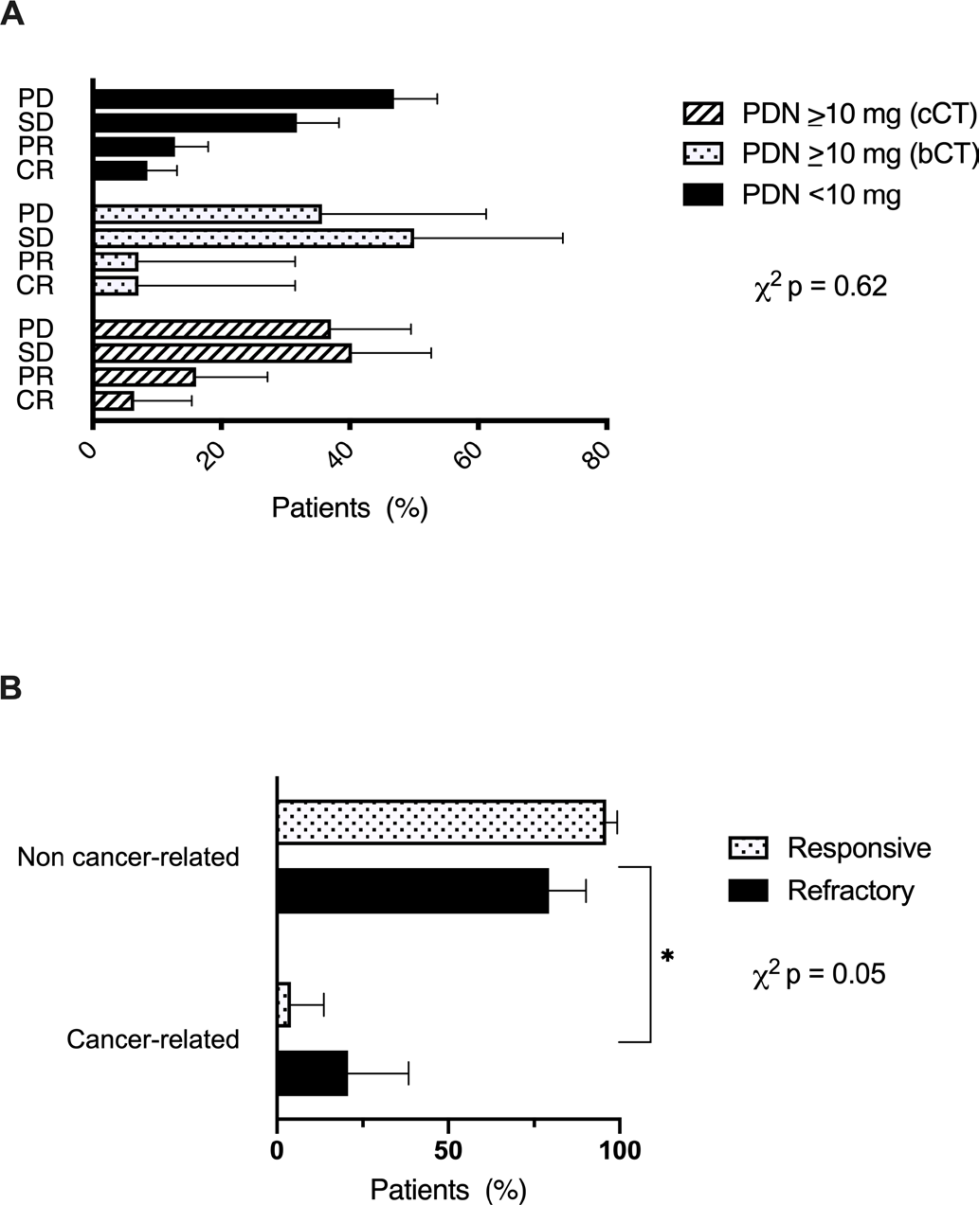
Relationship between timing of corticosteroid therapy (CT) (A), indication of CT (B) and response to immune checkpoint inhibitors (ICI) in hepatocellular carcinoma. Patients were defined as ICI responsive if they achieved complete response (CR), partial response (PR) or stable disease (SD) (n=160) as best overall response by RECIST version 1.1 criteria. Refractory patients were defined as those achieving progressive disease (PD) as best response to ICI (n=127). bCT, baseline corticosteroid therapy; cCT, concomitant corticosteroid therapy.

Next, we evaluated the distribution of radiologic responses across bCT, cCT or steroid-unexposed patients and found no significant difference across groups (p=0.62, figure 3A). Patients on CT for palliation of cancer-related symptoms were more likely to be ICI refractory (figure 3B, p=0.05), and have shorter median PFS (1.6 months, 95% CI 0.4 to 2.8 vs 8.8 months, 95% CI 4.2 to 13.5, p<0.01) and OS (4.9 months, 95% CI 0.5 to 14.1 vs 15.4 months, 95% CI 7.0 to 18.6, p=0.05, online supplemental figure 1 A–C) compared with cancer-unrelated indications.

Univariable analyses of survival revealed no difference in OS based on median duration of treatment (p=0.15) and median daily corticosteroid dose (p=0.75, online supplemental figure S2). In independent univariable and multivariable Cox regression models, neither bCT nor cCT predicted for OS or PFS (p>0.05, online supplemental tables S5–8). Analyses of OS and PFS in patients with Child-Pugh A cirrhosis were consistent with estimates observed in the whole patient population (online supplemental figures S3,4). We further tested the relationship between CT exposure and response, OS and PFS using post-landmark analysis to account for the time dependence of CT administration. In the first 3 months, 19 (1.6%) of the 62 evaluable patients with CT exposure during ICI had at least one course of corticosteroids. The median PFS and OS in these patients were 4.6 months (95% CI 1.4 to 9) and 7.5 months (2.5 to NR), respectively. The median PFS and OS in the subgroup of patients with CR+PR response were not reached. Succeeding timepoints (months 6, 9 and 12) showed longer PFS and OS durations for cCT compared with those without CT exposure (ie, 0 to 10 mg prednisone) or bCT (online supplemental table S9). When evaluating patients exposed to CT at any time point during ICI, PFS was 8.2 months (95% CI 5.6 to 12.5) while OS was 16.1 months (95% CI 8.9 to NR). Comparison of PFS and OS across the three groups of CT exposure did not show significant results (p>0.05).

## Discussion

We document for the first time that corticosteroid use alongside ICI is safe in patients with HCC, mirroring evidence in other indications.^16 17^ This is particularly reassuring for patients requiring cCT for irAE management, who, despite receiving higher doses of steroids for longer periods compared with other indications, had similar outcomes compared with steroid-unexposed patients. The low prevalence of bCT (5%), not predictive of outcome in our study, is perhaps unsurprising given that, unlike advanced lung cancer, unresectable HCC is a largely asymptomatic diagnosis and intracranial spread is rare.^18^ Interestingly, only patients with HCC receiving palliative CT had worse ORR, PFS and OS. This lends credence to the view that CT may correlate with prognosis by association with poor prognostic features (ie, cancer-related symptoms secondary to symptomatic or rapidly progressive disease) rather than blunting of ICI responsiveness.^19^

While limited by small sample size and lack of correlative analyses on peripheral immune cell responses following steroid treatment, the multi-center design of our study ensures adequate representation of the various etiologies of HCC and attempts to control for the diversity in clinical practice including corticosteroid prescribing. Given this study enrolled patients treated with ICI as part of routine clinical practice, our sample included patients with Child-Pugh B cirrhosis, largely excluded from clinical trials of ICI in HCC. While impaired liver function is a key prognostic determinant in HCC,^20^ this was unrelated to the provision of CT and supplementary analyses inclusive of Child-Pugh A patients only were concordant with main study outcomes (online supplemental figures S3–4). We could not ascertain the relationship between CT and comorbidities other than liver dysfunction, an aspect worth exploring in prospective studies.

To conclude, there is no sufficient evidence to suggest that CT exposure either prior to or during ICI therapy is associated with OS and PFS in patients with HCC; CT exposure does not associate with key clinicopathologic traits of HCC including stage, liver function, alpha-fetoprotein levels and line of therapy. CT for palliative indications identified patients with poorer response and survival from ICI and traces an interesting parallelism with evolving experience in lung cancer where association with adverse features rather than causality has emerged as a likely explanation of the detrimental role of CT.^19^ While mechanistic studies on the immune-modulatory effects of CT in ICI recipients are awaited, our clinical data are reassuring in suggesting that CT does not appear to worsen ORR and OS in HCC being treated with ICI. The relationship between CT and outcomes from combination regimens including ICI and anti-angiogenics should be further explored in prospective studies.

## References

[1] Flynn MJ, Sayed AA, Sharma R, et al Challenges and opportunities in the clinical development of immune checkpoint inhibitors for hepatocellular carcinoma. Hepatology 2019;69:2258–70. 10.1002/hep.30337 30382576

[2] El-Khoueiry AB, Sangro B, Yau T, et al Nivolumab in patients with advanced hepatocellular carcinoma (CheckMate 040): an open-label, non-comparative, phase 1/2 dose escalation and expansion trial. Lancet 2017;389:2492–502. 10.1016/S0140-6736(17)31046-2 28434648 PMC7539326

[3] Zhu AX, Finn RS, Edeline J, et al Pembrolizumab in patients with advanced hepatocellular carcinoma previously treated with sorafenib (KEYNOTE-224): a non-randomised, open-label phase 2 trial. Lancet Oncol 2018;19:940–52. 10.1016/S1470-2045(18)30351-6 29875066

[4] Yau T, Kang Y-K, Kim T-Y Nivolumab (NIVO)+ ipilimumab (IPI) combination therapy in patients (pts) with advanced hepatocellular carcinoma (aHCC): Results from CheckMate 040. Am J Clin Oncol 2019.

[5] Kudo M A new era in systemic therapy for hepatocellular carcinoma: atezolizumab plus bevacizumab combination therapy. Liver Cancer 2020;9:119–37. 10.1159/000505189 32399427 PMC7206602

[6] Pinato DJ, Howlett S, Ottaviani D, et al Association of prior antibiotic treatment with survival and response to immune checkpoint inhibitor therapy in patients with cancer. JAMA Oncol 2019;5:1774. 10.1001/jamaoncol.2019.2785 31513236 PMC6743060

[7] Rossi G, Pezzuto A, Sini C, et al Concomitant medications during immune checkpoint blockage in cancer patients: novel insights in this emerging clinical scenario. Crit Rev Oncol Hematol 2019;142:26–34. 10.1016/j.critrevonc.2019.07.005 31352168

[8] Miller S, McNutt L, McCann M-A, et al Use of corticosteroids for anorexia in palliative medicine: a systematic review. J Palliat Med 2014;17:482–5. 10.1089/jpm.2013.0324 24702642

[9] Michot JM, Bigenwald C, Champiat S, et al Immune-related adverse events with immune checkpoint blockade: a comprehensive review. Eur J Cancer 2016;54:139–48. 10.1016/j.ejca.2015.11.016 26765102

[10] Giles AJ, Hutchinson M-KND, Sonnemann HM, et al Dexamethasone-induced immunosuppression: mechanisms and implications for immunotherapy. J Immunother Cancer 2018;6:51. 10.1186/s40425-018-0371-5 29891009 PMC5996496

[11] Libert C, Dejager L How steroids steer T cells. Cell Rep 2014;7:938–9. 10.1016/j.celrep.2014.04.041 24856295

[12] Hussain N, Naeem M, Pinato DJ Concomitant medications and immune checkpoint inhibitor therapy for cancer: causation or association? Hum Vaccin Immunother 2020:1–7. 10.1080/21645515.2020.1769398 PMC7872020 32574106

[13] Arbour KC, Mezquita L, Long N, et al Impact of baseline steroids on efficacy of programmed cell death-1 and programmed death-ligand 1 blockade in patients with non-small-cell lung cancer. J Clin Oncol 2018;36:2872–8. 10.1200/JCO.2018.79.0006 30125216

[14] European Association for the Study of the Liver. Electronic address: easloffice@easloffice.eu, European Association for the Study of the Liver EASL clinical practice guidelines: management of hepatocellular carcinoma. J Hepatol 2018;69:182–236. 10.1016/j.jhep.2018.03.019 29628281

[15] Pinato DJ, Sharma R, Allara E, et al The ALBI grade provides objective hepatic reserve estimation across each BCLC stage of hepatocellular carcinoma. J Hepatol 2017;66:338–46. 10.1016/j.jhep.2016.09.008 27677714

[16] Santini FC, Rizvi H, Plodkowski AJ, et al Safety and efficacy of re-treating with immunotherapy after immune-related adverse events in patients with NSCLC. Cancer Immunol Res 2018;6:1093–9. 10.1158/2326-6066.CIR-17-0755 29991499 PMC6125223

[17] Horvat TZ, Adel NG, Dang T-O, et al Immune-related adverse events, need for systemic immunosuppression, and effects on survival and time to treatment failure in patients with melanoma treated with ipilimumab at Memorial Sloan Kettering Cancer Center. J Clin Oncol 2015;33:3193–8. 10.1200/JCO.2015.60.8448 26282644 PMC5087335

[18] Villanueva A Hepatocellular carcinoma. N Engl J Med 2019;380:1450–62. 10.1056/NEJMra1713263 30970190

[19] Ricciuti B, Dahlberg SE, Adeni A, et al Immune checkpoint inhibitor outcomes for patients with non-small-cell lung cancer receiving baseline corticosteroids for palliative versus nonpalliative indications. J Clin Oncol 2019;37:1927–34. 10.1200/JCO.19.00189 31206316

[20] Pinato DJ, Kaneko T, Saeed A, et al Immunotherapy in hepatocellular cancer patients with mild to severe liver dysfunction: adjunctive role of the ALBI grade. Cancers 2020;12. 10.3390/cancers12071862. [Epub ahead of print: 10 Jul 2020]. PMC7408648 32664319

