# Supplementary material for: Impact of corticosteroid therapy on the outcomes of hepatocellular carcinoma treated with immune checkpoint inhibitor therapy
Source: J Immunother Cancer. 2020 Oct 7;8(2):e000726. doi: 10.1136/jitc-2020-000726 (PMC7542664; doi:10.1136/jitc-2020-000726)

Supplement

Impact of corticosteroid therapy on the outcomes of hepatocellular carcinoma treated with immune checkpoint inhibitor therapy.

David J. Pinato et al.

Contents

Tables.

Supplementary Table 1. **Page 4.**
Geographical origin of HCC patients treated with ICI

Supplementary Table 2. **Page 4.**
The relationship between timing and indication of corticosteroid therapy.

Supplementary Table 3. **Page 5.**
Pairwise comparison of median duration of corticosteroid therapy (days) across indication.

Supplementary Table 4. **Page 5.**
Pairwise comparison of corticosteroid exposure expressed as median prednisone (PDN) equivalent dose in mg/day across indications.

Supplementary Table 5. **Page 6.**
Uni-variable analysis of factors contributing to overall survival.

Supplementary Table 6. **Page 7.**
Multivariable analysis of factors contributing to overall survival

Supplementary Table 7. **Page 8.**
Univariable analysis of factors contributing to progression-free survival.

Supplementary Table 8. **Page 9.**
Multivariable analysis of factors contributing to progression-free survival.

Supplementary Table 9. **Page 10.**
The relationship between corticosteroid exposure, response and survival:
landmark time survival analysis.

Figures.

Supplementary Figure 1. **Page 11.**
Kaplan-Meier curves illustrating the the relationship between indication for corticosteroid therapy (CT), OS and PFS.

Supplementary Figure 2. **Page 13.**
The relationship between duration of corticosteroid therapy (CT), daily predisone (PDN)-equivalent dose and OS

Supplementary Figure 3. **Page 14.**
The relationship between corticosteroid therapy (CT) OS (**A**) and PFS (**B**) in patients with Child Pugh A cirrhosis.

Supplementary Figure 4. **Page 15.**
The relationship between timing of corticosteroid therapy (CT) and OS (**A**) and PFS (**B**) in patients with Child Pugh A cirrhosis.

Table S1. Geographical Origin of HCC patients treated with ICI.

**Region,** Institution	**Patients** (%)
**Europe**	
Humanitas Cancer Center, Italy	37 (12)
Imperial College London, United Kingdom	6 (2)
Freiburg University, Germany	3 (1)
**North America**	
Mount Sinai, New York	93 (31)
MD Anderson Cancer Center, Texas	65 (21)
Kansas University Medical Center, Kansas	32 (10)
Weill Cornell Medical Center, New York	20 (7)
East Carolina Medical Centre, North Carolina	3 (1)
**Asia**	
Taipei Veterans General Hospital, Taiwan	45 (15)

Table S2. The relationship between timing and indication of corticosteroid therapy.

**Indication**	**bCT (%)**	**cCT (%)**	**p value**
Co-morbidity	0 (0)	6 (10)	**0.003***
Palliation	2 (27)	3 (5)	
irAE	0 (0)	27 (44)	
Procedure/Prophylaxis	12 (73)	25 (41)	
TOTAL	14 (100)	61 (100)	

Table S3. Pairwise comparison of median duration of corticosteroid therapy (days) across indication.

**Indications**	**Duration Indication 1 (Days) Median (IQR)**	**Duration Indication 2 (Days) Median (IQR)**	**Mann Whitney U**	**Adjusted§ p value (2-sided)**
Procedure/Prophylaxis vs Co-morbidity	2 (2)	5.5 (6)	8.18	**0.02***
Procedure/Prophylaxis vs Palliation	2 (2)	13.5 (9)	9.54	**0.01***
Procedure/Prophylaxis vs irAE	2 (2)	17 (6)	43.71	**<0.001***
Comorbidity vs Palliation	5.5 (6)	13.5 (9)	6.66	0.65
Comorbidity vs irAE	5.5 (6)	17 (6)	2.99	1.0
Palliation vs irAE	13.5 (9)	17 (6)	1	1.0

§ Significance values adjusted by the Bonferroni correction for multiple tests.

Table S4. Pairwise comparison of corticosteroid exposure expressed as median prednisone (PDN) equivalent dose in mg/day across indications.

**Indications**	**PDN equivalent dose Indication 1 Median (IQR)**	**PDN equivalent dose Indication 2 Median (IQR)**	**Mann Whitney U**	**Adjusted§ p value (2-sided)**
irAE vs Palliation	40 (45)	37.5 (47.5)	0.57	1.0
Palliation vs Procedure/Prophylaxis	37.5 (47.5)	50 (25)	4.11	1.0
irAE vs Comorbidity	40 (45)	67.5 (48.7)	0.83	1.0
irAE vs Procedure/Prophylaxis	40 (60)	50 (25)	11.3	**0.005***
Palliation vs Comorbidity	37.5 (47.5)	67.5 (48.7)	0.11	1.0
Procedure/Prophylaxis vs Comorbidity	50 (25)	67.5 (48.7)	12.6	**0.002***

§ Significance values adjusted by the Bonferroni correction for multiple tests.

Table S5. Uni-variable analysis of factors contributing to overall survival.

Predictor	Univariable model	
	**Hazard Ratio (95%CI)**	**P-value**
**Objective response rate (ORR)**<br>CR+PR/SD+PD	4.4 (2.5-7.8)	**<0.001***
**AFP**<br><400 ng/ml<br>>400 ng/ml	1.4 (1.0-1.9)	**0.03***
**BCLC Stage**<br>A-B<br>C	1.6 (1.1-2.5)	**0.02***
**Child Turcotte Pugh**<br>A/B	1.9 (1.3-2.6)	<0.001
**ECOG PS**<br>0-1/2	1.8 (0.9-3.5)	0.10
**Immunotherapy Treatment**<br>Monotherapy/<br>Combination	1.0 (0.6-1.8)	0.92
**Prior systemic therapy for HCC,**<br>0-1<br>>2	0.8 (0.4-1.6)	0.55
**Corticosteroid Therapy (any time)**<br>Y/N	0.8 (0.8-1.3)	0.88
**Corticosteroid Therapy prior to ICI***<br>Y/N	1.2 (0.5-2.3)	0.54
**Corticosteroid Therapy during ICI****<br>Y/N	0.8 (0.5-1.2)	0.29
**Indication for Corticosteroid Therapy**<br>PDN <10 mg/d<br>PDN ≥10 mg/d cancer-unrelated<br>PDN ≥10 mg/d cancer-related	-<br>0.8 (0.5-1.1)<br>1.7 (0.7-3.8)	-<br>0.20<br>0.23

*Defined as PDN ≥10 mg/d pre-ICI
**Defined as PDN ≥10 mg/d during ICI

Table S6. Multi-variable analysis of factors contributing to overall survival.

Predictor	Multivariable model 1		Multivariable model 2		Multivariable model 3	
	**Hazard Ratio (95%CI)**	**P-value**	**Hazard Ratio (95%CI)**	**P-value**	**Hazard Ratio (95%CI)**	**P-value**
**BCLC Stage**<br>A-B<br>C	1.4 (0.8-2.4)	0.13	1.5 (0.9-2.5)	0.13	1.4 (0.8-2.3)	0.19
**AFP**<br><400 ng/ml<br>>400 ng/ml	1.1 (0.7-1.8)	0.50	1.1 (0.8-1.7)	0.62	1.1 (0.7-1.7)	0.76
**Child Turcotte Pugh**<br>A/B	2.3 (1.4-3.5)	**<0.001***	2.2 (1.4-3.5)	**<0.001***	2.2 (1.4-3.5)	**<0.001***
**ECOG PS**<br>0-1/2	1.2 (0.5-2.7)	0.61	1.2 (0.6-2.7)	0.60	1.3 (0.6-2.9)	0.46
**Immunotherapy Treatment**<br>Monotherapy/<br>Combination	1.1 (0.6-2.0)	0.75	1.1 (0.6-1.9)	0.80	1.1 (0.6-2.0)	0.68
**Corticosteroid Therapy prior to ICI***<br>Y/N	3.3 (0.4-24.4)	0.23	-	-	-	-
**Corticosteroid Therapy during ICI****<br>Y/N	-	-	0.7 (0.4-1.3)	0.23	-	-
**Corticosteroid Therapy Indication**<br>PDN <10 mg/d<br>PDN ≥10 mg/d cancer-unrelated<br>PDN ≥10 mg/d cancer-related	-	-	-	-	 -<br>0.4 (0.2-0.9)<br>2.1 (0.9-5.3)	 **0.02***<br>**0.03***<br>0.09

Table S7. Uni-variable analysis of factors contributing to progression-free survival.

Predictor	Univariable model	
	**Hazard Ratio (95%CI)**	**P-value**
**BCLC Stage**		
A-B	1.4 (0.9-2.1)	0.06
C		
**AFP**	1.1 (0.8-1.5)	0.53
<400 ng/ml/		
>400 ng/ml		
**Child Turcotte Pugh**	1.2 (0.8-1.7)	0.32
A/B		
**ECOG PS**	2.3 (1.3-4.3)	**0.005***
0-1/2		
**Immunotherapy Treatment**	0.9 (0.5-1.6)	0.68
Monotherapy/		
Combination		
**Prior systemic therapy for HCC,**		
0-1	1.4 (0.8-2.4)	0.13
>2		
**Corticosteroid Therapy**	0.8 (0.6-1.2)	0.22
Y/N		
**Corticosteroid Therapy prior to ICI***		
Y/N	0.7 (0.3-1.5)	0.37
**Corticosteroid Therapy during ICI****		
Y/N	0.9 (0.6-1.3)	0.42
**Indication for Corticosteroid Therapy**		
PDN <10 mg/d	-	**0.001***
PDN ≥10 mg/d cancer-unrelated	0.7 (0.5-1.0)	0.07
PDN ≥10 mg/d cancer-related	3.4 (1.6-7.4)	**0.002***

Table S8. Multi-variable analysis of factors contributing to progression-free survival.

Predictor	Multivariable model 1		Multivariable model 2		Multivariable model 3	
	**Hazard Ratio (95%CI)**	**P-value**	**Hazard Ratio (95%CI)**	**P-value**	**Hazard Ratio (95%CI)**	**P-value**
**BCLC Stage**<br>A-B<br>C	1.0<br>(0.7-1.7)	0.70	1.1<br>(0.6-1.7)	0.75	1.1<br>(0.4-1.4)	0.74
**AFP**<br><400 ng/ml/<br>>400 ng/ml	1.0<br>(0.9-1.4)	0.75	0.9<br>(0.6-1.3)	0.57	0.8<br>(0.5-1.2)	0.44
**Immunotherapy Treatment**<br>Monotherapy/<br>Combination	0.7<br>(0.4-1.3)	0.35	0.7<br>(0.4-1.3)	0.29	0.7<br>(0.4-1.3)	0.33
**ECOG PS**<br>0-1/2	2.0<br>(1.0-4.0)	**0.03***	2.1<br>(1.1-4.0)	**0.03***	2.4<br>(1.2-4.6)	**0.001***
**Corticosteroid Therapy prior to ICI***<br>Y/N	3.9<br>(0.5-28.6)	0.17	-	-	-	-
**Corticosteroid Therapy during ICI****<br>Y/N	-	-	0.7<br>(0.4-1.2)	0.16	-	-
**Indication for Corticosteroid Therapy**<br>PDN <10 mg/d<br>PDN ≥10 mg/d cancer-unrelated<br>PDN ≥10 mg/d cancer-related	-	-	-	-	-<br>0.6 (0.3-1.0)<br>4.4 (1.7-11.1)	**0.001***<br>**0.05***<br>**0.002***

Table S9. The relationship between corticosteroid exposure, response and survival: landmark time survival analysis.

Corticosteroid (CT) Exposure	Time point of CT exposure (month)	Best Response	N	Death n (%)	Progression n (%)	Median PFS months (95% CI)	Median OS months (95% CI)
PDN >10 mg during ICI	first 3 mo.	CR + PR	1	0 (0)	0 (0)	NR	NR
		SD + PD	18	5 (28)	8 (44)	3.3 (1.4 – 12.1)	7.5 (2.5 – 9.0)
		All	19	5 (26)	8 (42)	4.6 (1.4 – 9)	7.5 (2.5 – NR)
	first 6 mo.	CR + PR	3	0 (0)	0 (0)	8.2 (7.5 – NR)	8.2 (7.5 – NR)
		SD + PD	24	7 (29)	13 (54)	4.6 (2.4 – 8.4)	8.5 (3.3 – 15.4)
		All	27	7 (26)	13 (48)	6.1 (2.5 – 8.4)	8.2 (7.1 – 9.9)
	first 9 mo.	CR + PR	3	2 (67)	3 (100)	8.2 (7.5 – NR)	8.2 (7.5 – NR)
		SD + PD	27	15 (56)	17 (63)	5.5 (2.5 – 8.4)	8.5 (4.1 – 10.4)
		All	30	17 (57)	20 (67)	6.3 (2.6 – 8.4)	8.2 (7.1 – 10.4)
	first 12 mo.	CR + PR	5	2 (40)	3 (60)	9.0 (7.5 – NR)	NR (7.5 – NR)
		SD + PD	27	17 (63)	17 (63)	5.5 (2.5 – 8.4)	8.5 (4.1 – 10.4)
		All	32	19 (59)	20 (62)	6.8 (3.3 – 9.0)	8.5 (7.3 – 12.8)
	any time*	CR + PR	14	3 (21)	4 (29)	NR (8.2 – NR)	NR (8.2 – NR)
		SD + PD	48	26 (54)	33 (69)	6.1 (3.1 – 8.9)	9.9 (7.2 – 17.7)
		All	62	29 (48)	37 (60)	8.2 (5.6 – 12.5)	16.1 (8.9 – NR)
PDN >10 mg prior to ICI		CR + PR	2	1 (50)	0 (0)	NR	12.8 (12.8 – NR)
		SD + PD	12	9 (75)	7 (58)	5.5 (1.4 – NR)	9.2 (2.7 – NR)
		All	14	10 (71)	7 (50)	6.8 (2.0 – NR)	10.4 (4.9 – 18)
PDN 0 to <10 mg**		CR + PR	45	10 (22)	11 (25)	NR (19.0 – NR)	23.3 (18.7 – NR)
		SD + PD	166	100 (60)	111 (67)	3.7 (3.2 – 4.2)	9.2 (7.3 – 11.7)
		All	211	110 (52)	122 (58)	4.8 (4.0 – 6.9)	12.3 (9.8 – 16.1)

Abbreviations: NR = Not reached; *2 patients without response evaluation; **15 patients without response evaluation

Corticosteroid Therapy in HCC

Figure S1. Kaplan-Meier curves illustrating the relationship between indication for corticosteroid therapy (CT) and OS (**A**). Panels B and C highlight the difference in PFS (**B**) and OS (**C**) between patients receiving corticosteroid therapy for cancer-related versus non cancer-related indications.

A.

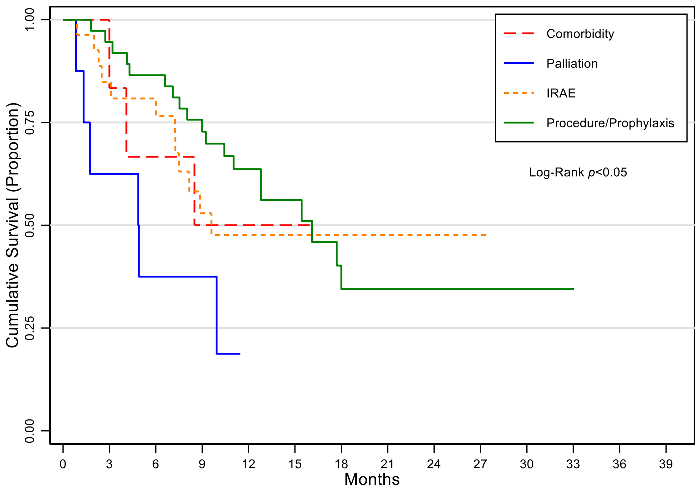

Number at risk	0	3	6	9	12	15	18	21	24	27	30	33	36	39
Comorbidity	6	6	4	3	2	1	0	0	0	0	0	0	0	0
Palliation	8	5	3	2	0	0	0	0	0	0	0	0	0	0
IRAE	27	22	19	10	9	7	5	4	1	1	0	0	0	0
Procedure/Prophylaxis	37	35	32	26	20	11	7	5	5	3	2	1	0	0

B.

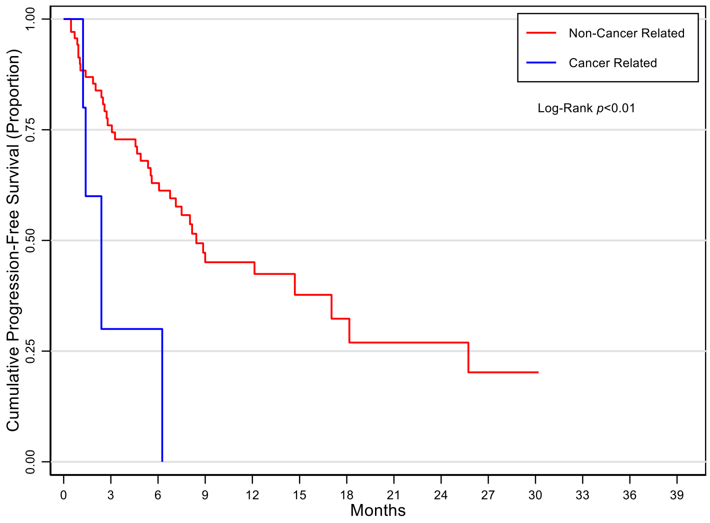

Number at risk	0	3	6	9	12	15	18	21	24	27	30	33	36	39
Non-Cancer Related	69	48	37	22	18	8	6	5	4	2	1	0	0	0
Cancer Related	5	1	1	0	0	0	0	0	0	0	0	0	0	0

C.

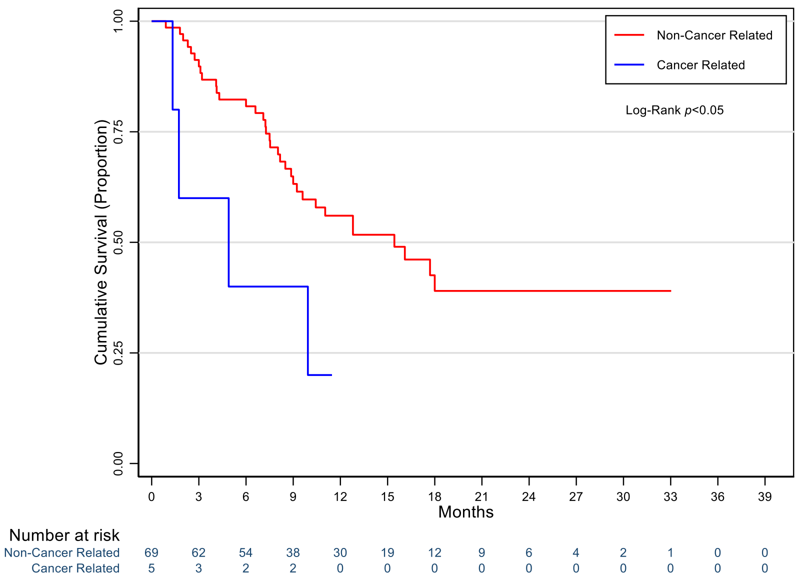
Non-Cancer Related
Cancer Related
Log-Rank *p*<0.05
Cumulative Survival (Proportion)
1.00
0.75
0.50
0.25
0.00
0
3
6
9
12
15
18
21
24
27
30
33
36
39
Months
Number at risk
Non-Cancer Related 69 62 54 38 30 19 12 9 6 4 2 1 0 0
Cancer Related 5 3 2 2 0 0 0 0 0 0 0 0 0 0

Figure S2. The relationship between duration of corticosteroid therapy (CT, **A**) daily predisone (PDN)-equivalent dose (**B**) and OS.

A.

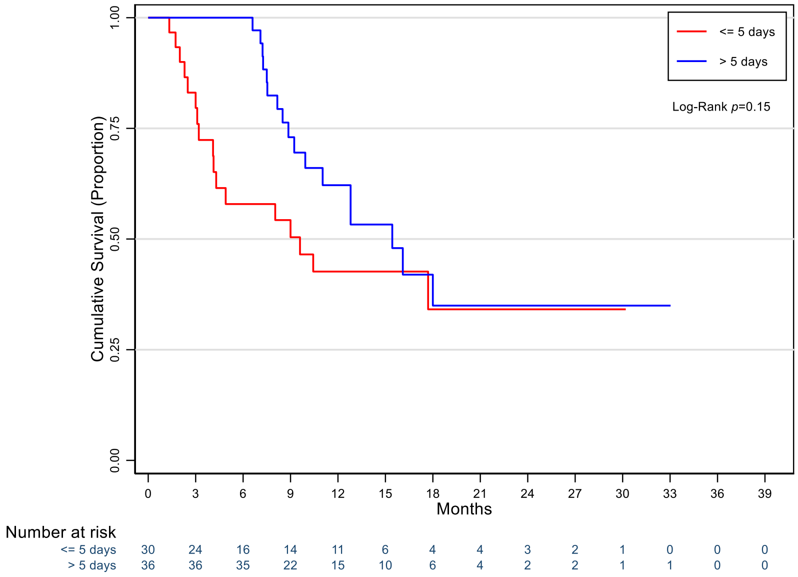

B.

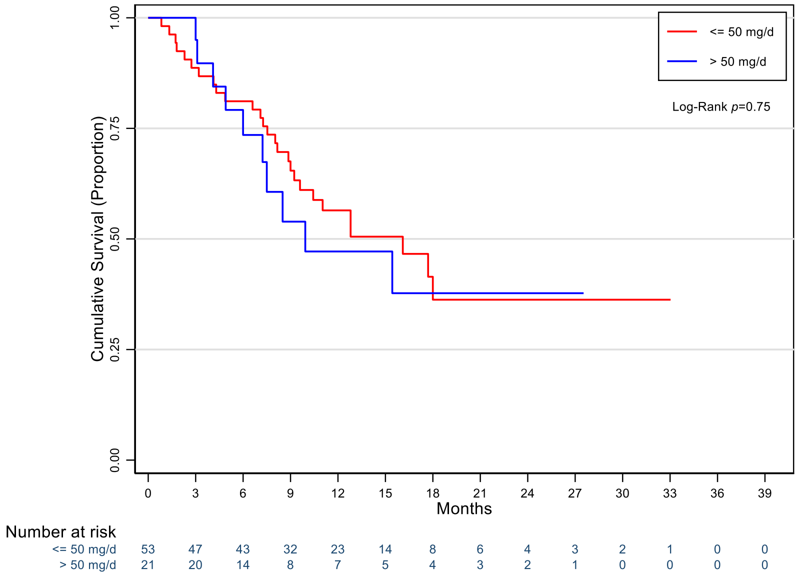

Figure S3. The relationship between corticosteroid therapy (CT) OS (**A**) and PFS (**B**) in patients with Child Pugh A cirrhosis.

A.

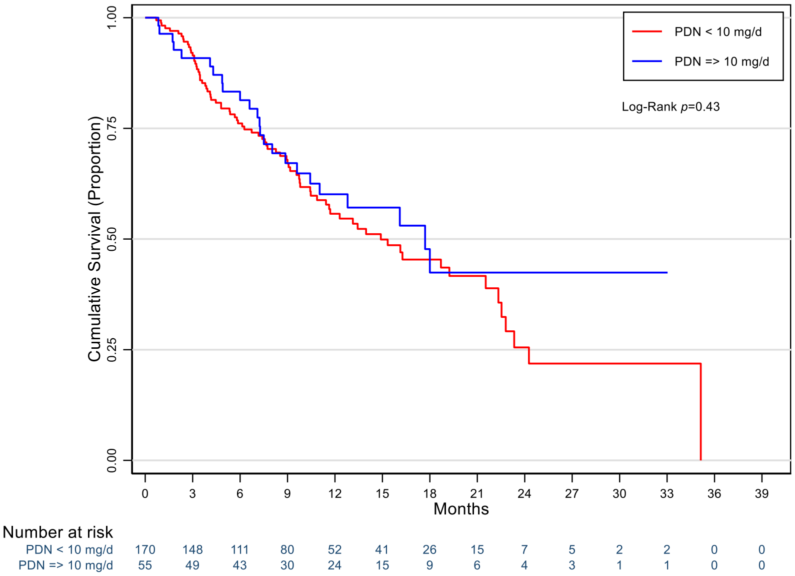

B.

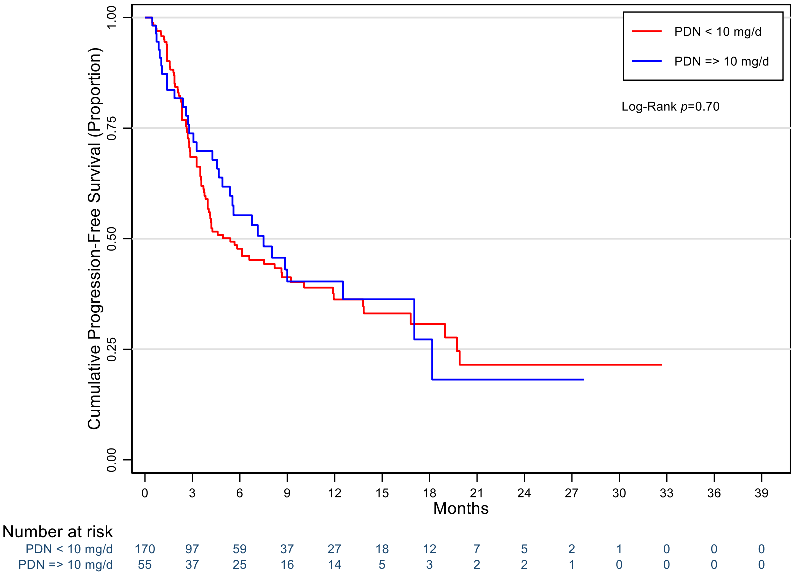

Pinato DJ, *et al. J Immunother Cancer* 2020; 8:e000726. doi: 10.1136/jitc-2020-000726

Figure S4. The relationship between timing of corticosteroid therapy (CT) and OS (**A**) and PFS (**B**) in patients with Child Pugh A cirrhosis.

A.

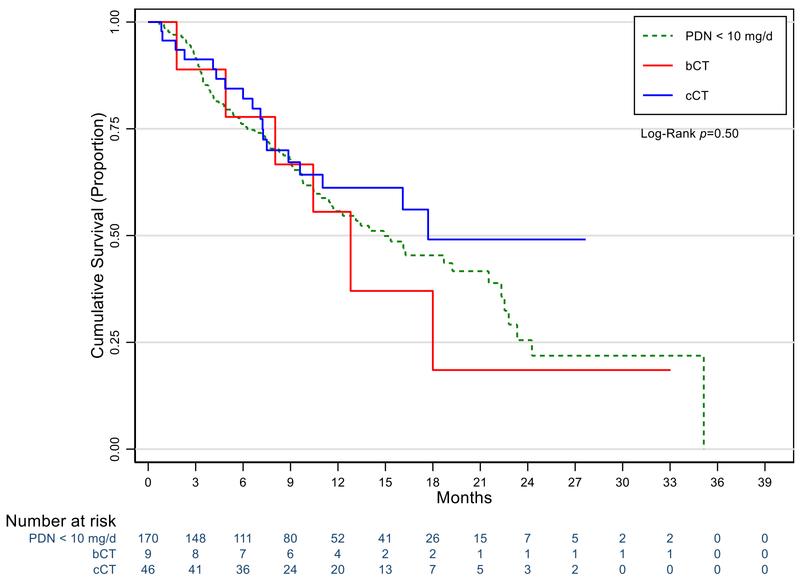

B.

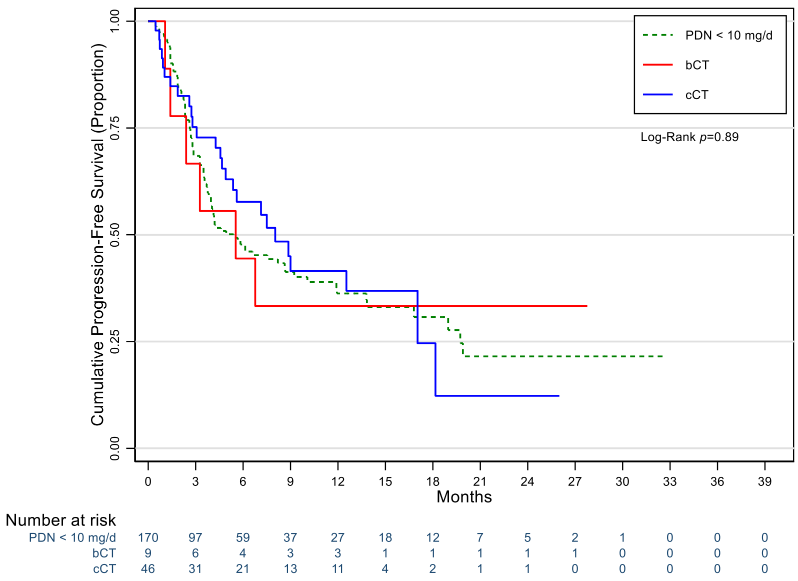

Supplement: Supplementary data [file jitc-2020-000726supp001.pdf]
